# Supplementary material for: Seizure-like behavior and hyperactivity in napb knockout zebrafish as a model for autism and epilepsy
Source: Sci Rep. 2025 Apr 29;15:14579. doi: 10.1038/s41598-025-96862-2 (PMC12041455; doi:10.1038/s41598-025-96862-2)
Supplement: Supplementary file 1 — Supplementary Material 1 [file 41598_2025_96862_MOESM1_ESM.pdf]

Supplementary Information

Seizure-like behavior and hyperactivity in *napb* knockout zebrafish: a model for autism and epilepsy

Kyung Chul Shin[1†], Waseem Hasan[2†], Gowher Ali[1], Doua Abdelrahman[2], Tala Abuarja[2], Lawrence W Stanton[1,3], Sahar I. Da'as[2,3*], Yongsoo Park[1,3*]

[1]Neurological Disorders Research Center, Qatar Biomedical Research Institute (QBRI), Hamad Bin Khalifa University (HBKU), Qatar Foundation, Doha, Qatar

[2]Department of Human Genetics, Sidra Medicine, Doha 26999, Qatar

[3]College of Health & Life Sciences (CHLS), Hamad Bin Khalifa University (HBKU), Qatar Foundation, Doha, Qatar

[†]These authors contributed equally to this work.

*Corresponding authors:

Dr. Yongsoo Park, Neurological Disorders Research Center, Qatar Biomedical Research Institute (QBRI), Hamad Bin Khalifa University (HBKU), Qatar Foundation, Doha, Qatar

E-mail: ypark@hbku.edu.qa

Dr. Sahar I. Da'as, Department of Human Genetics, Sidra Medicine, Doha 26999, Qatar

E-mail: sdaas@sidra.org

Supplementary Figure 1

NAPB zebrafish model. (**A**) Protein alignment between the human and zebrafish proteins using CLC sequence viewer software Qiagen. (**B**) High-Resolution Melting Analysis (HRM) to confirm the early CRISPR-induced indels. RNP mix was injected with fluorescein or rhodamine fluorescent dye in the one-cell stage embryos. At 24 hours post-fertilization (hpf), the injected embryos were screened for fluorescence. The extracted genomic DNA of pooled embryos was analyzed using heteroduplex protocol and High-Resolution Melting Analysis (HRM) to confirm the early CRISPR-induced indels in crispants. The HRM curve showed a distinctive curve between the crispants and controls. (**C**) Zebrafish model survival rate and developmental classification. NAPB model survival and developmental classification were calculated at 24 and 120 hpf, respectively; gene editing had no effect on survival rate nor gross morphology development. G1: severely affected development, G2: mildly affected development, G3: Normal development. (**D**) Injection of CRISPR RNP mix for gene editing at 1-cell stage mixed with Dextran, a fluorescein dye. The developing injected embryos show the distribution of the dye to confirm the injection.

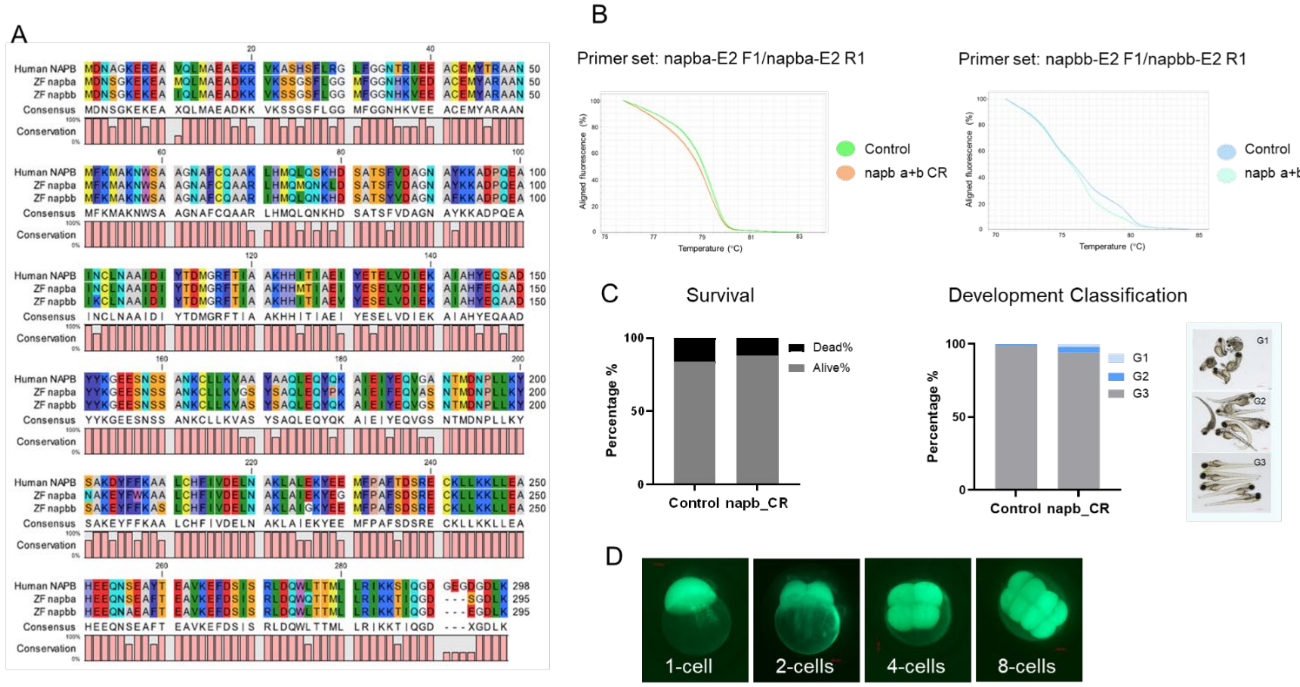

Supplementary Figure 2

Neuromuscular synaptogenesis of NAPB zebrafish model. (**A**) Representative images of Neuromuscular junctions at 120 hpf using Zeiss light sheet microscopy Z1. Immunofluorescence staining for presynaptic vesicle clusters (SV2; synaptic vesicle glycoprotein 2A, Green) and postsynaptic acetylcholine receptors (AChRs) with α-bungarotoxin (αBTX; postsynaptic, Red). Scale bar = 50 µm. (**B**) Colocalization of SV2-positive signal with αBTx using Mander's correlation coefficient (0 = no colocalization, 1 = full colocalization). A total of 11 larvae per group were subject to analysis. In violin plots, the thick line indicates the median and dotted lines indicate interquartile ranges. Welch unpair t-test was used to determine statistically significant differences.

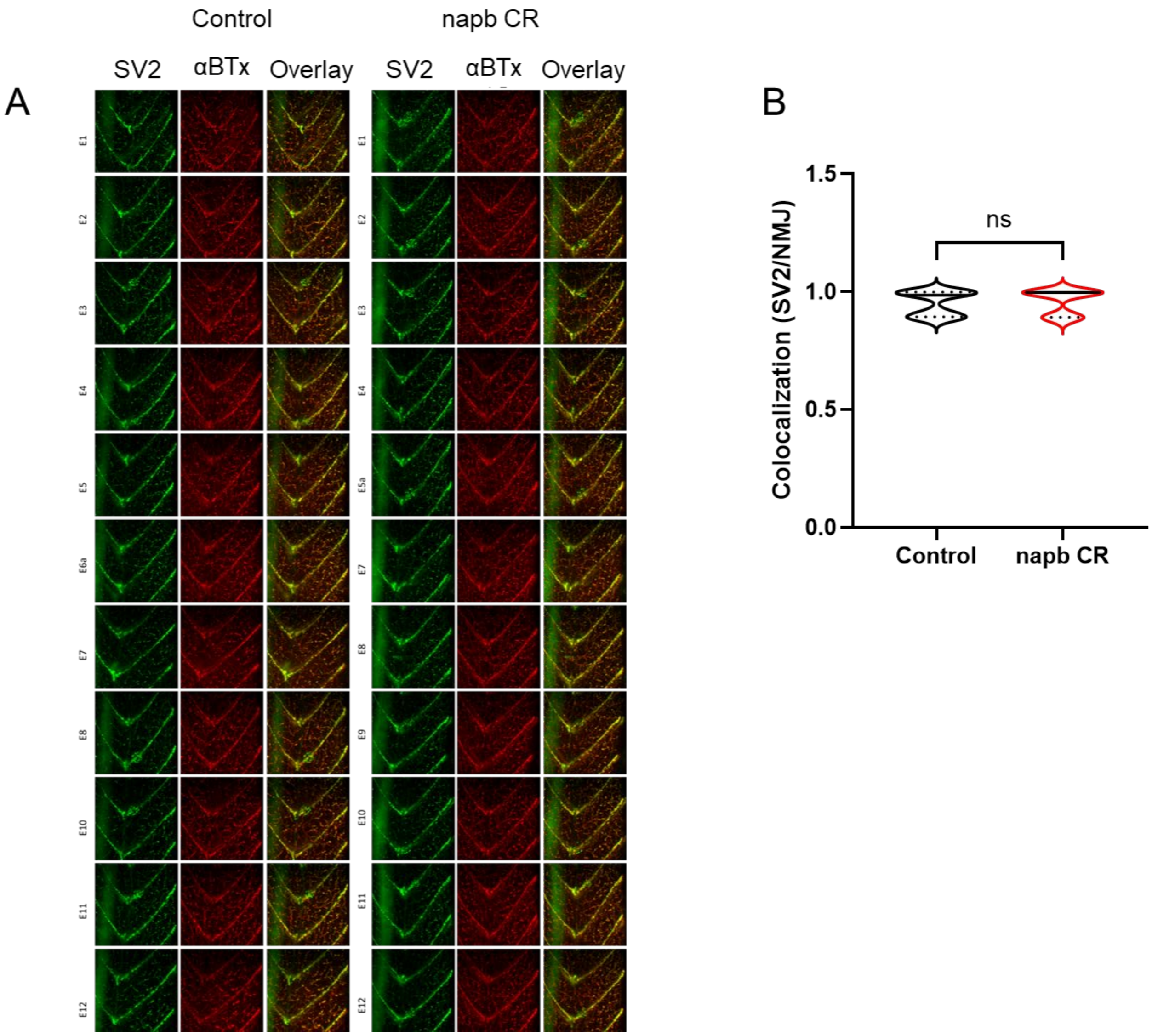

Supplementary Figure 3

Targeting *NAPB* zebrafish orthologs, *napba* and *napbb*. (**A**) The model survival rate was calculated at 24 hpf after targeting each ortholog separately by injecting the CRISPR RNP mix. (**B-D**) The locomotor behavior assay showed no significant impact for each ortholog separately, but when combined, the larvae demonstrated a significant change in locomotor behavior. Analysis of locomotor swimming behavior including total distance (**B**), swimming velocity (**C**), and rotation frequency (**D**). In violin plots, the thick line indicates the median and dotted lines indicate interquartile ranges. Welch and Brown-Forsythe's one-way ANOVA test was used to determine statistically significant differences. Control (n=230), *napb* CR (n=205), *napba* CR (n=24), and *napbb* CR (n=32).

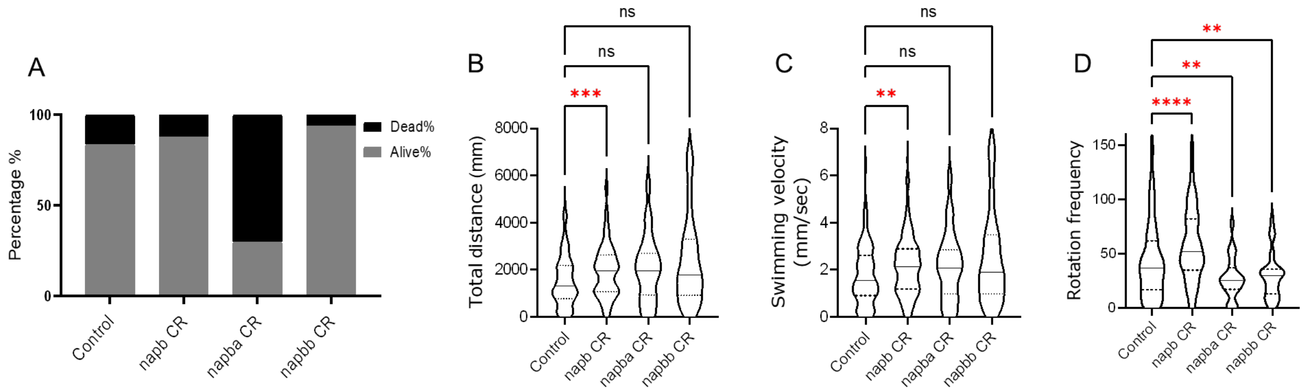

Supplementary Figure 4

Gene expression of GABA receptors in zebrafish larvae. (**A**) Quantification of relative mRNA expression levels using reverse transcription quantitative PCR (qPCR), with normalization to *actb1* (beta-actin) as the reference gene. Data are presented as means ± SD from two independent biological replicates. *actb1* (beta-actin), *gabra1* (GABA-A receptors subunit alpha-1), *gabra4* (GABA-A receptors subunit alpha-4), *gabrb1* (GABA-A receptors subunit beta-1), *gabrb2a* (GABA-A receptors subunit beta-2A), *gabrb3* (GABA-A receptors subunit beta-3), *gabrg2* (GABA-A receptors subunit gamma-2), and *gabrr1* (GABA-A receptors subunit rho-1).

A

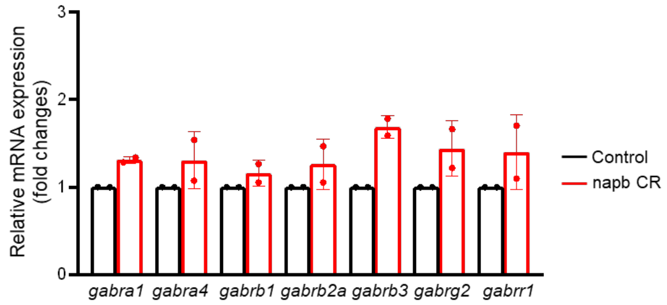

Supplementary Figure 5

Hyperactivity and seizure-like phenotype of *napb* CR. (**A-C**) The recording of the swimming locomotor activity through consecutive 12-min dark-light cycles. The analysis was calculated using Ethovision software (Noldus Technologies) and visualized as mean with standard error bars per 2-min time bins of dark-light cycles without PTZ or after 30 min of 15 mM PTZ treatment. (**A**) Total distance, (**B**) swimming velocity, and (**C**) rotation frequency. Welch unpair t-test was used to determine statistically significant differences.

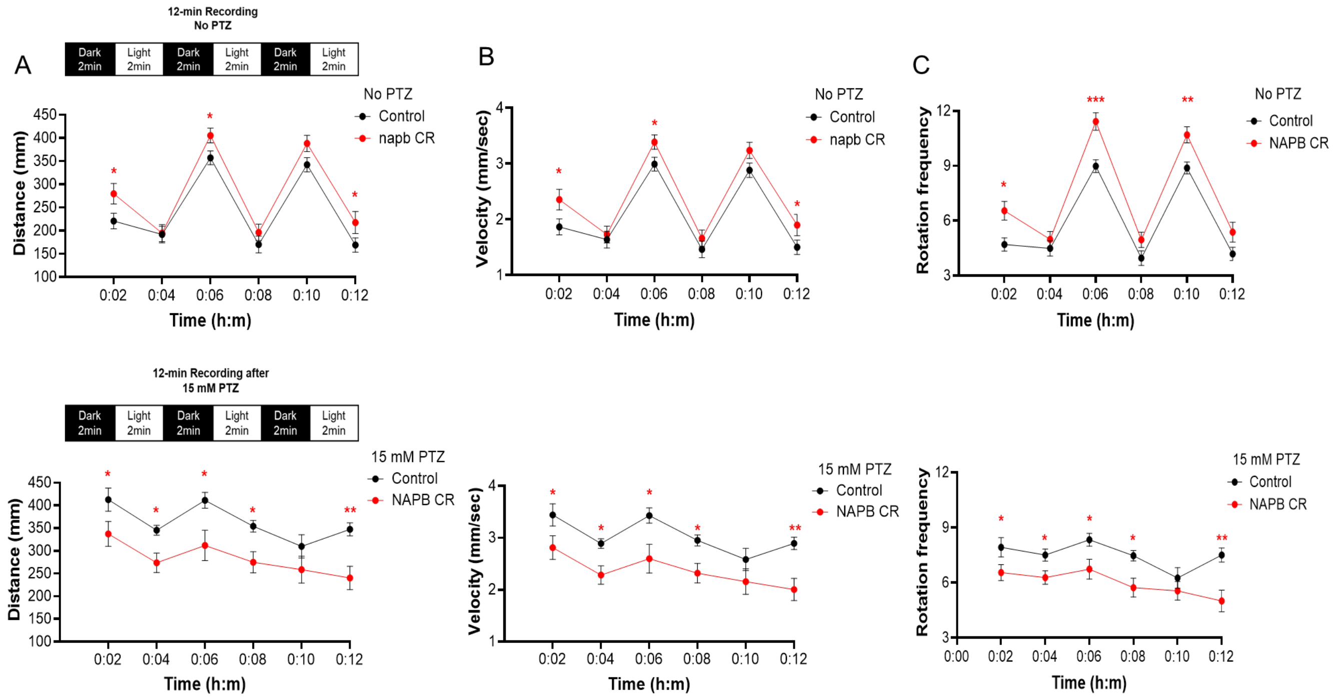

Supplementary Figure 6

Zebrafish *napb* CR locomotor responses during PTZ exposure. (**A-C**) The recording of the swimming locomotor activity in dark conditions. The analysis was presented as mean with standard error bars per 2 min-time bins during 30 min of 15 mM PTZ treatment. (**A**) Total distance, (**B**) swimming velocity, and (**C**) rotation frequency. Welch unpair t-test was used to determine statistically significant differences.

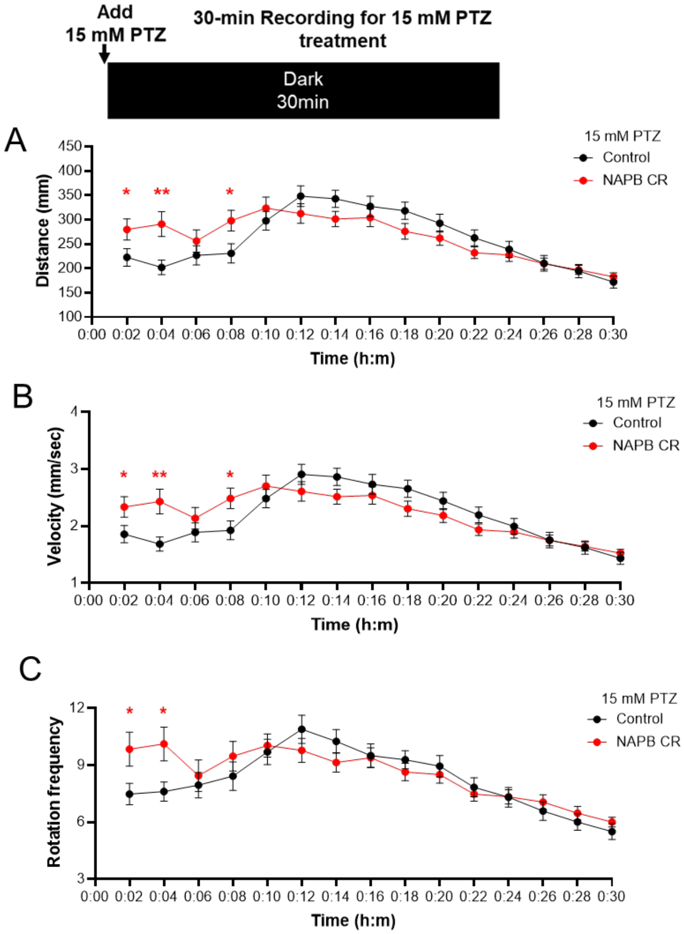

Supplementary Table 1. PCR primer sequence for *napba* and *napbb*

Primer name	Sequence
napbb-E5 F1	ggaaggtggctctccaggaaca
napbb-E5 R1	tcccccaactttaccactcagtg
napbb-E5 R1a	TTCTCGATGTCCACCAGCTCCG
napbb-E2 F1	gccatgcatctctgacttcaccc
napbb-E2 R1	ctcagatggcttgagggggtgt
napbb-E2 F1a	TCACAAAGTGGAGGAGGCTTGTG
napba-E1 F1	catcagcatcacccgacagcag
napba-E1 R1	CATCAGCTGCATGGCCTCCTTC
napba-E1 F1a w R1	tcgcctctcagccaaagagtgt
napba-R1a w F1	cacCCGAACATCCCTCCGAGAA
napba-E2 F1	gcattagacaggctacgacgcg
napba-E2 R1	tcacaccctggtcaccacagag
napba-E2 F1a	agGGGGAATCATAAGGTGGAGGA

Supplementary Table 2. Primer sequence for qPCR

Gene	NCBI accession number	Forward primer 5'-3'	Reverse primer 5'-3'	Product size (bp)
**qPCR**				
actb1	NM_131031.2	CTGGACTTTGAGCAGGAGATG	CAAGATTCCATACCCAGGAAGG	155
gabra1	NM_001077326.1	TTTGGCAGCTATGCCTACAC	TCTACGCTCTGTCCCATCAA	128
gabra4	NM_001017822.1	CCTGCATCATGACTGTAATCCT	GACGGTGGTGATTCCAAAGA	92
gabrb1	XM_002664133.5	AGCTGCCGCAATTCTCTATTA	ATCAGGGTTGAAGGCATGTAG	151
gabrb2a	NM_001024387.2	GCAATATGCCGGTGGTTAAAG	GTCAGGGTGTAGTCCATGTTT	160
gabrb3	XM_005166081.4	TTGATGTGGCGAGCATAGAC	GCTGGTCAGCTACTCTGTTATC	147
gabrg2	NM_001256250.1	TACGCCTCAACAGCAACAT	TGCGTCAATGGTCAACCTTA	161
gabrr1	NM_001025553.1	CTTACGGGTCACGGTTACATC	TGCAGAGATTTGTTCCCTCTC	147
