# Supplementary material for: Seizure-like behavior and hyperactivity in napb knockout zebrafish as a model for autism and epilepsy
Source: Sci Rep. 2025 Apr 29;15:14579. doi: 10.1038/s41598-025-96862-2 (PMC12041455; doi:10.1038/s41598-025-96862-2)
Supplement: Supplementary file 2 — Supplementary Material 2 [file 41598_2025_96862_MOESM2_ESM.pptx]

## Slide 1
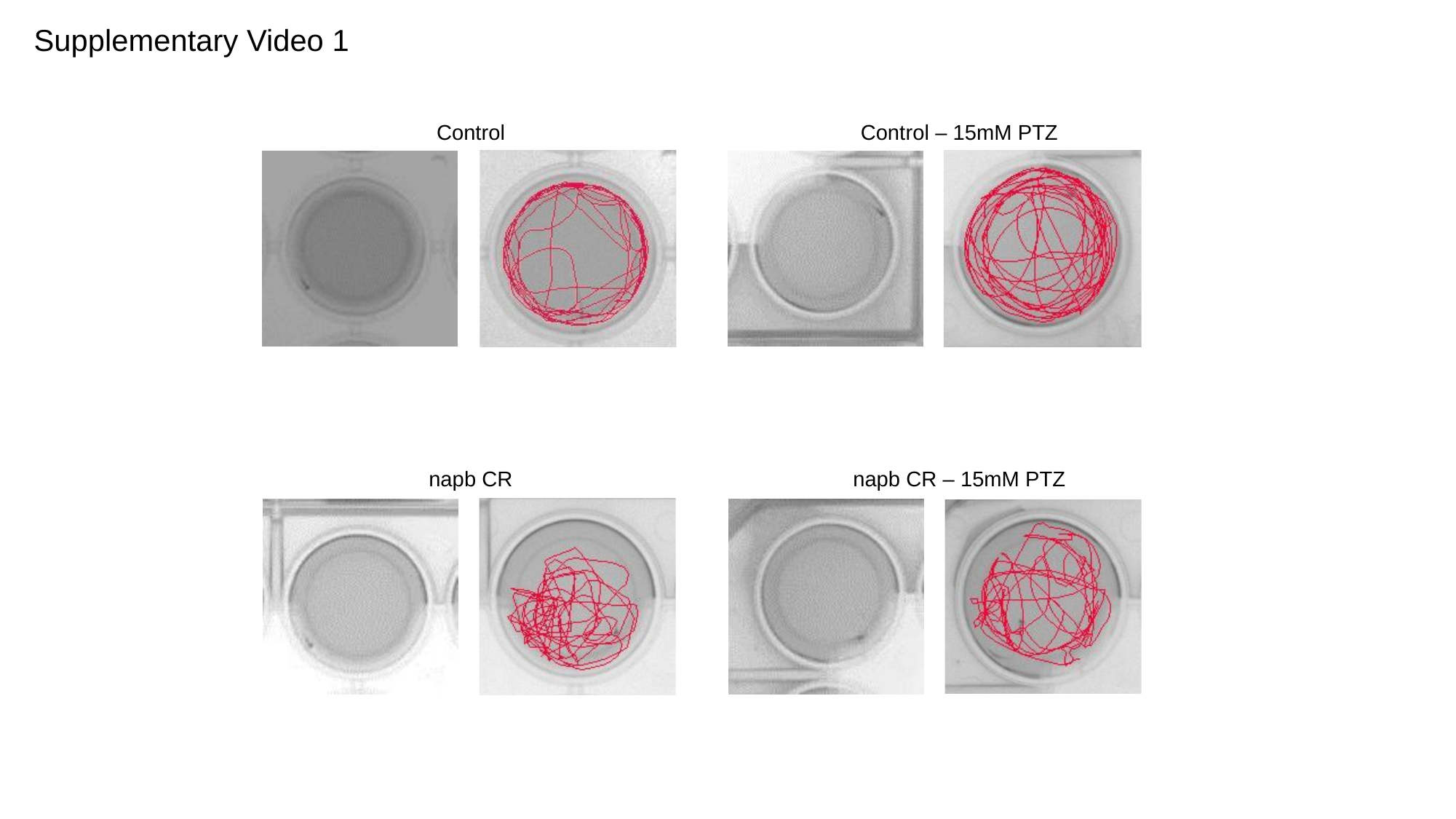

Supplementary Video 1
Control
Control – 15mM PTZ
napb CR
napb CR – 15mM PTZ

## Slide 2
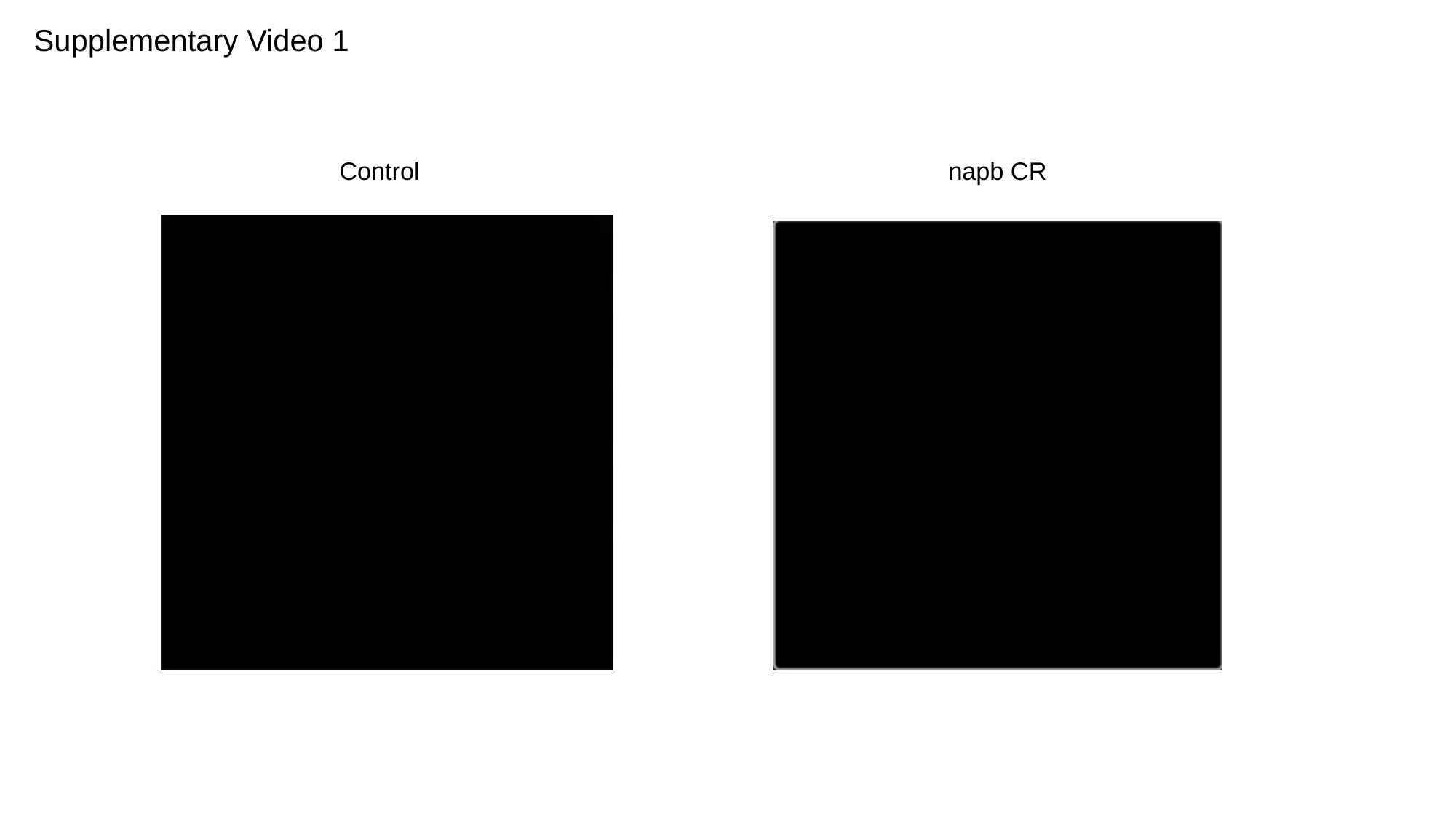

Supplementary Video 1
Control
napb CR
